# Impact of prenatal care provider on the use of ancillary health services during pregnancy

**DOI:** 10.1186/1471-2393-13-62

**Published:** 2013-03-11

**Authors:** Amy Metcalfe, Kristen Grabowska, Carol Weller, Suzanne C Tough

**Affiliations:** 1 Department of Obstetrics and Gynecology, University of British Columbia, BC Women’s and Children’s Hospital, 4500 Oak St, Vancouver, BC, V6H 3N1, Canada; 2 Division of Maternal Fetal Medicine, Department of Obstetrics and Gynecology, University of British Columbia, 13750 96 Avenue, Surrey, BC, V3V 1Z2, Canada; 3 Mental Health Screening and Early Identification, Addiction & Mental Health, Alberta Health Services, 10101 Southport Road SW, Calgary, AB, Canada; 4 Departments of Pediatrics and Community Health Sciences, University of Calgary Child Development Centre, c/o 2888 Shaganappi Trail NW, Calgary, AB, T3B 6A8, Canada

**Keywords:** Physician practice patterns, Pregnancy, Health services research

## Abstract

**Background:**

Recent declines in the provision of prenatal care by family physicians and the integration of midwives into the Canadian health care system have led to a shift in the pattern of prenatal care provision; however it is unknown if this also impacts use of other health services during pregnancy. This study aimed to assess the impact of the type of prenatal care provider on the self-reported use of ancillary services during pregnancy.

**Methods:**

Data for this study was obtained from the *All Our Babies* study, a community-based prospective cohort study of women’s experiences during pregnancy and the post-partum period. Chi-square tests and logistic regression were used to assess the association between type of prenatal care provider and use of ancillary health services in pregnancy.

**Results:**

During pregnancy, 85.8% of women reported accessing ancillary health services. Compared to women who received prenatal care from a family physician, women who saw a midwife were less likely to call a nurse telephone advice line (OR = 0.30, 95% CI: 0.18-0.50) and visit the emergency department (OR = 0.47, 95% CI: 0.24-0.89), but were more likely receive chiropractic care (OR = 4.07, 95% CI: 2.49-6.67). Women who received their prenatal care from an obstetrician were more likely to visit a walk-in clinic (OR = 1.51, 95% CI: 1.11-2.05) than those who were cared for by a family physician.

**Conclusions:**

Prenatal care is a complex entity and referral pathways between care providers and services are not always clear. This can lead to the provision of fragmented care and create opportunities for errors and loss of information. All types of care providers have a role in addressing the full range of health needs that pregnant women experience.

## Background

Prenatal care is a publically funded health service available to all Canadian women; however, the provision of prenatal care in Canada is changing as a result of a decrease in the proportion of family physicians who provide prenatal and intra-partum care and the introduction of public funding of midwifery in some Canadian provinces [1-4]. Yet it is unknown how, or if, changes in prenatal care provision for low-risk pregnant women affect use of other ancillary services. The impact on what Chan refers to as ‘the declining comprehensiveness of primary care’ on other parts of the health care system is unknown [3]. Although, Coco hypothesized that the reduction in the number of family physicians providing prenatal care may impact access to ancillary services for women, such as use of walk-in clinics, visits to other health professionals, and calls to nurse-telephone advice lines [5]. If non-obstetric issues are not routinely addressed in the context of prenatal care or are treated in an inconsistent manner through disjointed episodes of care in walk-in clinics and emergency departments, this may lead to increased costs to the health care system due to duplication of services and the provision of inconsistent health information from multiple care providers. A smooth transition between care providers has been identified as an aspect of quality prenatal care [6], yet studies have shown that multiple prenatal care providers give different information to women, which results in confusion and anxiety [7].

The literature indicates that family physicians, midwives and obstetricians provide different intra-partum services to their patients, with obstetricians more frequently using medical and surgical interventions such as labour induction, episiotomies, assisted vaginal deliveries and cesearean sections [8-15]. Despite these different practice styles, few appreciable differences are seen in birth outcomes for women with low risk pregnancies [1,9,13,14,16]. Bai et al. postulate that obstetricians may be more likely to intervene as they are alert to managing women with high risk pregnancies [8], while Matthias asserts that practice differences are the result of different underlying philosophies about how risky birth can be [11]. While much work has focused on understanding reasons for, and implications of, intra-partum practice pattern differences, few studies report on the impact of practice pattern variation in a prenatal setting. While the number and frequency of prenatal visits for low-risk patients is common across specialties, the number of contact hours differs with typical prenatal visits for uncomplicated patients taking approximately 30–45 minutes with a midwife, compared to 15 minutes with family physicians and 10 minutes with obstetricians [17]. Another study shows that prenatal care provided by midwives tends to be less medicalized than care provided by physicians [6].

This study aimed to assess the impact of the type of prenatal care provider (family physician vs. midwife vs. obstetrician) on the self-reported use of ancillary services during pregnancy for a group of women at low medical risk. To our knowledge, this is the first study to evaluate the relationship between the type of prenatal care provider on service use during pregnancy other than for prenatal care in the context of a publically funded health care system.

## Methods

### Study participants

The All Our Babies study is a prospective community-based cohort study of women’s experiences during pregnancy and the early post-partum period. Women were recruited from physician’s offices, posters in the community and through follow-up phone calls after receiving pregnancy-related blood work. Women were eligible to participate if they were able to communicate in English, were accessing prenatal care in Calgary, Alberta, Canada and were less than 24 weeks pregnant. Women were asked to complete three written questionnaires – one prior to 24 weeks of gestation, one between 34 and 36 weeks of gestation, and one at 4 months post-partum. Questionnaires consisted of validated scales and investigator derived questions. Following verbal consent to participate, participants were sent a package that included an information letter, a written consent form, a contact information form, the questionnaire and a postage-paid self-addressed return envelope. Participants who did not return their questionnaires within three weeks were contacted by telephone and email to remind them to complete the questionnaire and to provide them the opportunity to complete the questionnaire over the phone or to be mailed another questionnaire. Participants received a gift card to a grocery store for completion of each questionnaire and were sent congratulation cards following the birth of their baby and semi-annual newsletters to promote continual engagement with the study. Data were collected between May 2008 and May 2010 following pilot testing and revision of the questionnaires for clarity. This study was approved by the Conjoint Health Research Ethics Board at the University of Calgary and all participants provided written informed consent prior to participation. Please see http://www.ucalgary.ca/stough/allourbabies and McDonald et al. [18] for additional information on questionnaire content and survey methods or Appendix 1 for specific questions used in this analysis.

Overall, 1654 women participated in the All Our Babies cohort with a retention rate of 81%. Individuals with multiple gestation pregnancies (n = 24), who delivered preterm (gestational age <37 weeks) (n = 70), who had a caesarean delivery (n = 270), and who did not complete all three questionnaires (n = 383) were excluded from this analysis leaving a final sample size of 907. Completion of all three questionnaires was essential for this research question, as each questionnaire asked about the type of prenatal care provider and use of ancillary health services during pregnancy.

### Type of prenatal care provider

Prenatal care in Calgary is typically provided by family physicians practicing alone or in the context of a low-risk maternity care clinic, although women can also self-refer to a midwife to obtain prenatal care. Since April 2009, midwifery has been publically funded in Alberta; prior to this, if women wanted to access midwifery care they were required to pay out of pocket [1,2].

The majority of midwives in Alberta practice in community-based settings and attend to women at low medical risk. Many women are seen by their own family physician early in pregnancy and are then referred to a low risk maternity clinic later in gestation. Women typically require a referral to see an obstetrician, and if referred, this transition of care would typically occur in the late second trimester.

Women were asked on each questionnaire from whom they received prenatal care – a family physician in an appointment-based office, a family physician in a low risk maternity clinic, an obstetrician, a midwife, a walk-in clinic physician or other. Women were categorized as having an obstetrician as their prenatal care provider if they ever selected this option. They were categorized as seeing a midwife if they ever reported this option and did not report seeing an obstetrician. They were categorized as having another prenatal care provider if they never reported seeing an obstetrician, a midwife, a family physician in an appointment-based office, or a family physician from a low risk maternity clinic. The remaining women were categorized as having their prenatal care provided by a family physician practicing either in an appointment-based office or in a low-risk maternity clinic. It was not possible to confirm the accuracy of women’s assessments of their type of prenatal care provider or which type of care provider was the ‘main’ prenatal care provider for women who reported seeing multiple types of prenatal care providers.

### Use of ancillary health services during pregnancy

For the purposes of this study the term ‘ancillary health services’ was defined as health services accessed during pregnancy for purposes other than regular prenatal care. On each survey, participants were asked if they had accessed any of the following services for any reason other than for prenatal care, and the number of visits to each service: chiropractor, emergency department, family physician, nurse telephone advice line (Telehealth), nutritionist/dietician, physiotherapist, psychologist/psychiatrist, social worker, specialist physician (other than an obstetrician), walk-in clinic or any other health care provider. They were also asked if they had been hospitalized overnight. Please see Table 1 for a brief overview of how women can access these services.

**Table 1:** Access to ancillary services

**Type of service**	**Referral type**	**Cost**
Chiropractor	Self-referral or practitioner-referral	Chiropractic care is not covered by provincial health insurance. Patients can pay out of pocket or through a supplemental health insurance plan.
Emergency Department	Self-referral or practitioner-referral	Cost is covered by provincial health insurance.
Family Physician	Self-referral or practitioner-referral	Cost is covered by provincial health insurance.
Hospitalized Overnight	Practitioner-referral	Cost is covered by provincial health insurance.
Nurse Telephone Advice Line	Self-referral	Cost is covered by provincial health insurance.
Nutritionist/Dietician	Self-referral or practitioner-referral	Most dietary counseling is not covered by provincial health insurance. Patients can pay out of pocket or through a supplemental health insurance plan.
Physiotherapist	Self-referral or practitioner-referral	Most physiotherapy is not covered by provincial health insurance. Patients can pay out of pocket or through a supplemental health insurance plan.
Psychologist/Psychiatrist	Practitioner-referral	Costs for a psychiatrist are covered by provincial health insurance. Patients can pay out of pocket or through a supplemental health insurance plan to see a psychologist.
Social Worker	Practitioner-referral	Cost is covered by government.
Specialist Physician (other than an obstetrician)	Practitioner-referral	Cost is covered by provincial health insurance.
Walk-In Clinic	Self-referral	Cost is covered by provincial health insurance.

### Statistical analysis

Descriptive statistics were used to characterize the sample. Chi-square tests and the Kruskal-Wallis test were used to assess for differences in demographic factors. Unadjusted logistic regression models were applied to the data first; these contained only the variable related to type of prenatal care provider. Subsequently, multivariable logistic regression models that also contained demographic (maternal age, education, ethnicity) and pregnancy-related (gravidity, use of prescription medications) covariates. Likelihood ratio tests were significant (p < 0.001) for all fully-adjusted models indicating that the covariates were significant predictors of the outcome of interest. All analyses were conducted using Stata SE Version 11.

Two sensitivity analyses were conducted. The first examined ancillary service utilization early (prior to 24 weeks of gestation) and late (after 32 weeks of gestation) to determine if different patterns of ancillary resource use were present as the frequency of contact with prenatal care providers increases late in gestation. It was hypothesized that increased contact with prenatal care providers late in gestation might decrease use of ancillary services. The second sensitivity analysis limited the sample to women who reported only having a single type of care provider throughout their pregnancy as it was hypothesized that women who received the majority of their prenatal care from a family physician or midwife but were referred to an obstetrician later in pregnancy might have other medical risk factors that would influence ancillary service use.

## Results

### Type of prenatal care provider

Overall, 536 women (59.1%) received their prenatal care from a family physician or a low-risk maternity clinic; 286 (31.5%) saw an obstetrician; 82 (9.0%) saw a midwife; and 3 (0.3%) received their prenatal care from another source (i.e. walk-in clinic). Due to the low proportion of women who received their prenatal care from another source, these participants have been removed from all further analyses. Two hundred and seventy-one women (22.0%) reported seeing multiple prenatal care providers throughout the course of their pregnancy and were categorized as receiving prenatal care from a family physician, midwife or obstetrician as outlined in the methods section.

### Participant characteristics

As seen in Table 2, the majority of women had a partner, were born in Canada, have completed post-secondary education and had an annual household income of at least $60,000. Women did not differ across groups, except women who received their prenatal care from a midwife were significantly more likely to be Caucasian (p = 0.03). Women who participated in the All Our Babies cohort are representative of the pregnant population in Canada in terms of parity, pre-pregnancy BMI, and number of prenatal care visits; however, women in the All Our Babies study tend to be slightly older, better educated and have a higher household income [18].

**Table 2:** Participant characteristics

**Characteristic**	**Type of prenatal care provider**			**p-value**
	**Family physician**	**Midwife**	**Obstetrician**	
	**N = 536**	**N = 82**	**N = 286**	
	**N (%)**	**N (%)**	**N (%)**	
Maternal Age ≥35 years	94 (18.2)	15 (18.5)	61 (21.9)	0.45
Previously been pregnant	322 (60.1)	56 (68.2)	186 (65.0)	0.19
Currently has a partner	532 (99.4)	82 (100.0)	284 (99.3)	0.75
Caucasian Ethnicity	415 (77.7)	74 (90.2)	220 (76.9)	**0.03**
Born in Canada	419 (78.3)	69 (84.1)	216 (75.5)	0.24
Completed Post-Secondary Education	427 (80.0)	63 (76.8)	224 (78.3)	0.74
Currently working or attending school	345 (64.4)	49 (59.8)	169 (59.1)	0.29
Annual Household Income ≥ $60,000	438 (84.9)	68 (85.0)	234 (84.5)	0.99
Own their home	434 (81.0)	69 (84.1)	221 (77.3)	0.32

### Contact with prenatal care providers

While the median number of prenatal visits was not significantly different between groups (p = 0.37), participants who received their prenatal care from an obstetrician were significantly more likely to have less than 6 prenatal visits, the minimum number of prenatal visits recommended by the Society of Obstetricians and Gynecologists of Canada [19] and Health Canada’s Guidelines for Family-Centred Maternity and Newborn Care [20], throughout the course of their pregnancy (5.6%) compared to participants who received their prenatal care from a family physician (2.4%) or a midwife (2.4%) (p = 0.03). Participants who received their prenatal care from a midwife were significantly more likely to have their first prenatal visit in the first trimester (97.6%), than participants who received their prenatal care from a family physician (85.4%) or an obstetrician (88.1%) (p = 0.007).

### Use of ancillary health services during pregnancy

Seven hundred and seventy nine women (85.8%) reported using at least one ancillary service during the course of their pregnancy, and this did not differ based on the type of prenatal care provider (p = 0.88). Women who received their prenatal care from a midwife accessed fewer types of ancillary services (median 2, range 0–7) than women who accessed their prenatal care from a family physician (median 3, range 0–11) or an obstetrician (median 3, range 0–9) (p = 0.04).

Women who received their prenatal care from an obstetrician were significantly more likely to visit a walk-in clinic than women who received their prenatal care from a family physician (OR = 1.51, 95% CI: 1.11-2.05) (Table 3). Women who received their prenatal care from a midwife were significantly less likely to go to the emergency department (OR = 0.47, 95% CI: 0.24-0.89) or call a telephone nurse advice line (OR = 0.30, 95% CI: 0.18-0.50) compared to women who received their prenatal care from a family physician, and were significantly more likely to visit a chiropractor (OR = 4.07, 95% CI: 2.49-6.67) (Table 3).

**Table 3:** Use of ancillary health services during pregnancy

**Type of ancillary health service**	**Type of prenatal care provider**							
	**Family physician**		**Midwife**			**Obstetrician**		
	**N = 536**		**N = 82**			**N = 286**		
	**N (%)**	**OR (95% CI)**	**N (%)**	**Unadjusted OR (95% CI) *p value***	**Adjusted OR (95% CI) *p value***	**N (%)**	**Unadjusted OR (95% CI) *p value***	**Adjusted OR (95% CI) *p value***
Chiropractor	139 (25.9)	1.0 (ref)	48 (58.5)	**4.03 (2.50-6.52) *p < 0.001***	**4.07 (2.49-6.67) *p < 0.001***	64 (22.4)	0.82 (0.59-1.16) *p = 0.26*	0.80 (0.56-1.13) *p = 0.21*
Emergency Department	150 (28.0)	1.0 (ref)	12 (14.6)	**0.44 (0.23-0.84)*p = 0.01***	**0.47 (0.24-0.89) *p = 0.02***	98 (34.3)	1.34 (0.99-1.83) *p = 0.06*	1.31 (0.96-1.80) *p = 0.09*
Family Physician	387 (72.2)	1.0 (ref)	57 (69.5)	0.88 (0.53-1.46) *p = 0.61*	0.96 (0.57-1.61) *p = 0.88*	204 (71.3)	0.96 (0.70-1.32) *p = 0.79*	0.92 (0.66-1.27) *p = 0.61*
Hospitalized Overnight	28 (5.2)	1.0 (ref)	5 (6.1)	1.18 (0.44-3.14) *p = 0.74*	1.24 (0.46-3.36) *p = 0.67*	20 (7.0)	1.36 (0.75-2.47) *p = 0.30*	1.29 (0.70-2.38) *p = 0.41*
Nurse Telephone Advice Line	322 (60.1)	1.0 (ref)	25 (30.5)	**0.29 (0.18-0.48) *p < 0.001***	**0.30 (0.18-0.50) *p < 0.001***	179 (62.6)	1.11 (0.83-1.49) *p = 0.48*	1.15 (0.84-1.55) *p = 0.38*
Nutritionist/Dietician	38 (7.1)	1.0 (ref)	5 (6.1)	0.85 (0.32-2.23) *p = 0.74*	1.00 (0.38-2.68) *p = 0.99*	26 (9.1)	1.31 (0.78-2.21) *p = 0.31*	1.30 (0.76-2.21) *p = 0.34*
Physiotherapist	48 (9.0)	1.0 (ref)	9 (11.0)	1.25 (0.59-2.66) *p = 0.56*	1.32 (0.59-2.95) *p = 0.50*	21 (7.3)	0.81 (0.47-1.37) *p = 0.43*	0.80 (0.46-1.40) *p = 0.43*
Psychologist/Psychiatrist	44 (8.2)	1.0 (ref)	6 (7.3)	0.88 (0.36-2.14) *p = 0.78*	0.99 (0.40-2.43) *p = 0.98*	22 (7.7)	0.93 (0.55-1.59) *p = 0.80*	0.92 (0.53-1.59) *p = 0.77*
Social Worker	15 (2.8)	1.0 (ref)	1 (1.2)	0.43 (0.06-3.29) *p = 0.42*	0.55 (0.07-4.34) *p = 0.57*	14 (4.9)	1.79 (0.85-3.76) *p = 0.13*	1.58 (0.74-3.39) *p = 0.24*
Specialist Physician (other than an obstetrician)	97 (18.1)	1.0 (ref)	10 (12.2)	0.63 (0.31-1.26) *p = 0.19*	0.66 (0.33-1.34) *p = 0.25*	63 (22.0)	1.28 (0.90-1.82) *p = 0.18*	1.27 (0.88-1.82) *p = 0.20*
Walk-In Clinic	178 (33.2)	1.0 (ref)	20 (24.4)	0.65 (0.38-1.11) *p = 0.11*	0.74 (0.43-1.28) *p = 0.28*	119 (41.6)	**1.43 (1.07-1.93) *p = 0.02***	**1.51 (1.11-2.05) *p = 0.01***

* Analyses are adjusted for maternal age, education, ethnicity, gravidity and use of prescription medications during pregnancy.

### Sensitivity analysis

Two sensitivity analyses were conducted. The first examined health resource utilization early and late in gestation; the results were in the same direction as those presented in Table 3, hence only the overall findings are presented. The same pattern of service utilization early and late in gestation, irrespective of the type of prenatal care provider, suggests that ancillary service use is not influenced by the frequency of contact with the prenatal care provider. The second sensitivity analysis examined health resource utilization only among women who had a single type of prenatal care provider. The results were also in the same direction as the overall findings, but the relationship between seeing an obstetrician and visiting the emergency department became statistically significant (OR = 1.51, 95% CI 1.03-2.21), suggesting that women who were attended to exclusively by an obstetrician may have other medical risk factors necessitating use of emergency services compared to women who may have received a single consultation from an obstetrician and received the bulk of their prenatal care from a midwife or family physician.

## Discussion

This study of low-risk pregnant women, who were largely cared for by physicians (less than 10% of women received their prenatal care from a midwife), shows that regardless of prenatal care provider, the majority of women access at least one ancillary service during the course of their pregnancy. This speaks to the opportunity for coordination of care across multiple services and settings to ensure that women receive consistent information, to avoid duplication of services, and to assist prenatal care providers in attending to the broader needs of pregnant women. Statistically significant differences were seen for some, but not all, ancillary services based on the type of prenatal care provider. Despite restriction to a medically low-risk population and statistical adjustment for baseline differences between groups and for pregnancy-related factors that might predict service utilization such as gravidity and the use of prescription medications, it cannot be ascertained whether these differences are the result of different practice styles of midwives, family physicians and obstetricians or if they are due to unmeasured maternal factors that are associated with the type of prenatal care provider they may choose or, in some cases, require. Alternatively, these findings may suggest that many women who are pregnant will still present with a gestalt of health-related needs which need to be addressed or monitored throughout pregnancy.

Our findings with regards to the association between the type of prenatal care provider and the number of prenatal care visits and the timing of first prenatal visit are comparable to those reported in the Maternity Experiences Survey [13]. O’Brien et al. found that women who received their prenatal care from a midwife were more likely to access care early and had more prenatal visits [13]. A qualitative study examining midwifery care concluded that women who seek midwifery care wanted to pursue a more ‘natural approach’ to pregnancy and childbirth [21]. This same study found that women who chose midwifery desired to distance themselves from biomedical interventions [21]. The National Maternity Care Attitudes Study found that nulliparous women who received their prenatal care from obstetricians, midwives, and family physician represented different populations of women in terms of both demographic characteristics and attitudes towards the use of medical interventions during labour and delivery [22]. While few demographic differences were observed in this study, attitudinal differences may be reflected in our findings, as women who received their prenatal care from a midwife were not any less likely to access ancillary services but they were less likely to access biomedical services provided through emergency departments and nurse telephone advice lines and more likely to access complementary and alternative medicine (CAM) resources such as chiropractic care. CAM use in Canada is common; 12.4% of the general population reporting that they had accessed at least one CAM service in the past year, with chiropractic care being the fourth most common type of CAM service used (following massage, acupuncture and homeopathy) [23]. While women have been found to be twice as likely to assess CAM services in Canada as men [23], population-based estimates of CAM use in the context of pregnancy are lacking. A study comprising women who contacted the Motherisk program (a teratogen information service based in Toronto Ontario Canada) found that approximately 60% of women used CAM treatments to alleviate nausea and vomiting in pregnancy [24]. A Quebec study examining the use of herbal products in pregnancy estimated that 9% of pregnant women used an herbal supplement alone or in combination with prescription medications [25]. Differences in participant recruitment strategies and baseline characteristics likely explain some, but not all, of the differences in these rates. Unfortunately data on use of CAM services other than chiropractic care was not available in the current study; understanding the prevalence, predictors and outcome of CAM use during pregnancy is an important area for future research.

Women who obtained their prenatal care from an obstetrician would typically have been referred to this level of care due to higher underlying medical or fetal risk than is typically seen in the population of pregnant women cared for by family physicians. Attempts were made to restrict this sample to women at low medical risk; however, these women may have had underlying medical or obstetrical risk factors that influenced their use of ancillary services, particularly walk-in clinics and the emergency department. Additionally, it is possible that some visits classified as emergency department visits were actually obstetrical triage visits, thereby supporting a hypothesis of possible increased risk status in this population. After 20 weeks of gestation, labour and delivery triage is done on the obstetrical wards and not in the emergency room, even though women might initially present at the emergency room [26]. Alternatively, increased use of walk-in clinics and the emergency department may be related to the availability of after-hours pregnancy-related care. This should be explored in future studies. The consistency of findings between the main analysis that includes women who ever saw an obstetrician for prenatal care and the sensitivity analysis that examined women who only saw an obstetrician for prenatal care also supports the hypothesis of increased medical and/or fetal risk in this group.

As midwifery becomes more established, it is likely that the proportion of women choosing this option for prenatal care will increase. Data from the Canadian Maternity Experiences Survey suggests that women who receive their prenatal care from midwives are more likely to be over the age of 35, have completed post-secondary education and to have used folic acid before pregnancy compared to women who receive their prenatal care from obstetricians or family physicians [13]. Midwives were publicly funded in Alberta as of April 1, 2009 (midway through the recruitment period for this study) [1]. Other studies have found an increase in the use of midwifery services once these services were insured [1]. The increased capacity of midwives to provide prenatal and intra-partum care will be important, as it is estimated that only 11% of family physicians in Canada provide intra-partum care [27], and of those who do not currently offer this service to their patients, 25% have indicated that nothing could convince them to provide intra-partum care [4].

This trend of high resource utilization and accessing multiple unrelated services during pregnancy is likely to continue, regardless of the type of prenatal care provider, as the proportion of pregnancies in older women and women with underlying medical disease continues to increase [2,28]. This study points to the need for increased coordination of care as women see multiple health professionals throughout their pregnancies both for their prenatal care and for ancillary services, who may be providing them with conflicting advice. Yet, despite the increased risk for complications in these pregnancies [29], few, if any, guidelines exist for Canadian prenatal care providers to help guide them in when their patients should be referred to other ancillary services in pregnancy. The Society of Obstetricians and Gynecologists of Canada’s ‘Guidelines for Care During Pregnancy and Childbirth’ contains no references to when women should be referred to other sources of care [19]. While the Health Canada Guidelines for Family-Centred Maternity and Newborn Care speak to the need for appropriate referral to other services and outline that there could potentially be many types of care providers involved in the provision of prenatal care, they stop short of indicating when a referral should be made and what services pregnant women should, or should not, be referred to [20].

### Limitations

There are limitations to this study. All service use is self-reported; however, as this sample is limited to women with vaginal delivery and appropriate length of gestation, and this data was collected prospectively, the possibility of recall bias is minimized. Unfortunately, data was not collected on whether ancillary services use was related to pregnancy or not, merely whether it occurred in the prenatal period, so we cannot determine if women were self-referring to ancillary care services or were going at the recommendation of their prenatal care provider. This is important as many of the ancillary services examined in this study would not require referral; thus observed differences may be related to the type of prenatal care provider women chose, and not because of the type of prenatal care received from different providers. This study was limited to women living in a single province during a transition period from regulated midwifery to regulated and provincially reimbursed midwifery. It is unknown if these results can be generalized to other provinces where midwifery is more established. Misclassification bias is also possible if women were unable to accurately report who their prenatal care provider was. We presume that women would be most likely to report that they saw an obstetrician, when they really saw a family physician, which would bias the results towards the null. Misclassification is also possible for the 22% of women who reported seeing multiple types of prenatal care providers; for example, a woman who received the majority of her prenatal care from a family physician and had a single visit to an obstetrician would be classified in this study as receiving her prenatal care from an obstetrician. However, as our findings were robust when limited to women with a single type of care provider, this is unlikely to change our conclusions. Additionally, this study attempted to recruit women from the general population of pregnant women (the majority of whom can be classified as low medical risk) and efforts were made to further restrict this analysis to women with uncomplicated pregnancies; however, residual confounding by pregnancy-related or general medical complications is possible. This may be particularly relevant for women whose primary prenatal care provider was an obstetrician, who typically would have been referred to this level of care provider.

## Conclusion

In conclusion, understanding what ancillary care services women access during pregnancy is an important step in measuring women’s access to non-obstetric services during pregnancy. Prenatal care is a complex entity and referral pathways between care providers and services are not always clear. This can lead to the provision of fragmented care and create opportunities for errors and loss of information. All types of care providers have a role in addressing the full range of health needs that pregnant women experience. Future studies should examine the referral patterns of prenatal care providers and the content of prenatal care in relation to ancillary service use

## Appendix 1. Selected Questions from the All Our Babies Questionnaires

### From Questionnaire 1 (<24 weeks of gestation)

Which of the following health care providers did you see for your first prenatal visit?

• A walk-in clinic doctor

• A family doctor in an appointment based office

• A doctor in a Low Risk Maternity Clinic

• An obstetrician

• A midwife

• Other

Approximately how many weeks pregnant were you for your first prenatal care visit? Your best guess is ok. Each month has approximately 4 weeks. For example, if you are 2 and a half months pregnant, you are approximately 10 weeks pregnant.

____Weeks

Between the time you found out you were pregnant and now, have you visited any of the following for any reason? How many times each? Indicate all that apply.

• Visited a family doctor (for reasons other than a regular prenatal visit):

• Number of family doctor visits: ____

• Visited a walk-in clinic doctor

• Number of walk-in clinic visits: ____

• Visited a specialist physician

• Number of specialist visits: ____

• Visited the hospital Emergency Department

• Number of ER visits: ____

• Stayed overnight in a hospital

• Number of nights in hospital: ____

• Saw a physiotherapist

• Number of physiotherapist visits: ____

• Saw a chiropractor

• Number of chiropractor visits: ____

• Saw a psychologist or psychiatrist

• Number of psychologist visits: ____

• Saw a nutritionist/dietician

• Number of nutritionist visits: ____

• Saw a social worker

• Number of social worker visits: ____

• Called Healthlink, the Calgary Health Region 24-hour help line

• Number of Healthlink calls: ____

• Saw any other type of health care provider(s)

• No visits to healthcare providers

Have you ever been pregnant before?

• Yes

• No

Do you rent or own the housing you are currently living in?

• Rent

• Own

• Living with family (no rent)

• Other

How would you describe your current marital status?

• Single

• Divorced

• Single with partner

• Separated

• Married

• Widowed

• Common law

What is your birth date?

_____________ (MM DD YYYY)

What is the highest level of education you have completed?

• Some Elementary or High School (Grades 1–12)

• Graduated High School

• Some college, trade, university

• Graduated college, trade, university

• Some graduate school

• Completed graduate school

Were you born in Canada?

• Yes

• No

How would you describe your ethnic background?

• White/Caucasian

• Filipino

• Latin American

• Black/African North American

• First Nations person registered (under the Indian Act of Canada)

• Southeast Asian

• First Nations person not registered

• Arab

• Inuit

• West Asian

• Métis

• Korean

• Chinese

• Japanese

• South Asian

• Mixed/Other:

What is the total income, before taxes and deductions, of all household members from all sources in the past 12 months? Your best guess is ok.

• Less than $10,000

• $10,000 -$19,999

• $20,000 -$29,999

• $30,000 -$39,999

• $40,000 -$49,999

• $50,000 -$59,999

• $60,000 -$69,999

• $70,000 -$79,999

• $80,000 -$89,999

• $90,000 -$99,999

• $100,000 or more

### From Questionnaire 2 (34–36 weeks of gestation)

For your prenatal care have you seen… Select all that apply.

• A walk-in clinic doctor

• A family doctor in an appointment based office

• A doctor in a Low Risk Maternity Clinic

• An obstetrician

• A midwife

• Other

During this pregnancy, have you taken any prescription medications?

• Yes

• No

Between the time you found out you were pregnant and now, have you visited any of the following for any reason? How many times each? Indicate all that apply.

• Visited a family doctor (for reasons other than a regular prenatal visit):

• Number of family doctor visits: ____

• Visited a walk-in clinic doctor

• Number of walk-in clinic visits: ____

• Visited a specialist physician

• Number of specialist visits: ____

• Visited the hospital Emergency Department

• Number of ER visits: ____

• Stayed overnight in a hospital

• Number of nights in hospital: ____

• Saw a physiotherapist

• Number of physiotherapist visits: ____

• Saw a chiropractor

• Number of chiropractor visits: ____

• Saw a psychologist or psychiatrist

• Number of psychologist visits: ____

• Saw a nutritionist/dietician

• Number of nutritionist visits: ____

• Saw a social worker

• Number of social worker visits: ____

• Called Healthlink, the Calgary Health Region 24-hour help line

• Number of Healthlink calls: ____

• Saw any other type of health care provider(s)

• No visits to healthcare providers

Which of the following best describes your MAIN activity? Please select only one.

• Working at a job or business (self-employed, part-time, full-time)

• A homemaker

• Looking for a job

• On maternity leave

• A student

• On medical leave

• Other

### Questionnaire 3 (4 months post-partum)

How many weeks pregnant were you when your baby/babies was/were born?

____Weeks

For your prenatal care did you see… Select all that apply.

• A walk-in clinic doctor

• A family doctor in an appointment based office

• A doctor in a Low Risk Maternity Clinic

• An obstetrician

• A midwife

• Other

Including all prenatal care providers, approximately how many prenatal visits did you have in total during your pregnancy?

____Visits

Between the last questionnaire and now (you would have been in your last trimester), have you visited any of the following for any reason? How many times each?

• Visited a family doctor (for reasons other than a regular prenatal visit):

• Number of family doctor visits: ____

• Visited a walk-in clinic doctor

• Number of walk-in clinic visits: ____

• Visited a specialist physician

• Number of specialist visits: ____

• Visited the hospital Emergency Department

• Number of ER visits: ____

• Stayed overnight in a hospital

• Number of nights in hospital: ____

• Saw a physiotherapist

• Number of physiotherapist visits: ____

• Saw a chiropractor

• Number of chiropractor visits: ____

• Saw a psychologist or psychiatrist

• Number of psychologist visits: ____

• Saw a nutritionist/dietician

• Number of nutritionist visits: ____

• Saw a social worker

• Number of social worker visits: ____

• Called Healthlink, the Calgary Health Region 24-hour help line

• Number of Healthlink calls: ____

• Saw any other type of health care provider(s)

• No visits to healthcare providers

## Competing interests

None of the authors have any competing interests to declare.

## Authors’ contributions

ST designed the overall study and secured funding. AM designed this sub-study, conducted the analysis and drafted the manuscript. All authors participated in the interpretation of findings; critically reviewed the manuscript and approved the final version for submission.

## Pre-publication history

The pre-publication history for this paper can be accessed here:

http://www.biomedcentral.com/1471-2393/13/62/prepub
